# Machine learning-based prediction of fainting during blood donations using donor properties and weather data as features

**DOI:** 10.1186/s12911-022-01971-x

**Published:** 2022-08-20

**Authors:** Susanne Suessner, Norbert Niklas, Ulrich Bodenhofer, Jens Meier

**Affiliations:** 1 Red Cross Transfusion Service of Upper Austria, Krankenhausstraße 7, 4010 Linz, Austria; 2 School of Informatics, Communications, and Media, University of Applied Sciences Upper Austria, Softwarepark 11, 4232 Hagenberg, Austria; 3 Department for Anesthesiology and Critical Care, Kepler University Clinic, Kepler University Linz, Krankenhausstraße 9, 4010 Linz, Austria; 4 Department of Anesthesiology and Intensive Care Medicine, Kepler University Clinic, Johannes Kepler University, Altenberger Strasse 69, 4040 Linz, Austria

**Keywords:** Blood donation, Fainting, Donor safety

## Abstract

**Background and objectives:**

Fainting is a well-known side effect of blood donation. Such adverse experiences can diminish the return rate for further blood donations. Identifying factors associated with fainting could help prevent adverse incidents during blood donation.

**Materials and methods:**

Data of 85,040 blood donations from whole blood and apheresis donors within four consecutive years were included in this retrospective study. Seven different machine learning models (random forests, artificial neural networks, XGradient Boosting, AdaBoost, logistic regression, K nearest neighbors, and support vector machines) for predicting fainting during blood donation were established. The used features derived from the data obtained from the questionnaire every donor has to fill in before the donation and weather data of the day of the donation.

**Results:**

One thousand seven hundred fifteen fainting reactions were observed in 228 846 blood donations from 88,003 donors over a study period of 48 months. Similar values for all machine learning algorithms investigated for NPV, PPV, AUC, and F1-score were obtained. In general, NPV was above 0.996, whereas PPV was below 0.03. AUC and F1-score were close to 0.9 for all models. Essential features predicting fainting during blood donation were systolic and diastolic blood pressure and ambient temperature, humidity, and barometric pressure.

**Conclusion:**

Machine-learning algorithms can establish prediction models of fainting in blood donors. These new tools can reduce adverse reactions during blood donation and improve donor safety and minimize negative associations relating to blood donation.

## Introduction

Healthy, unpaid blood donors guarantee the availability of sufficient blood components for transfusion, which play an essential role in modern medical care. Altruism is one of the main motivation factors for voluntary unpaid blood donation [1]. However, blood donation is not only associated with positive effects such as a reduction of cardiovascular events in blood donors [2], an increase in high-density lipoprotein [3], or a general feeling of satisfaction [4]. Still, it can also result in anemia and iron deficiency in the donor [5, 6]. Prevalence rates of up to 10% of negative experiences are reported with blood donation [7]. Hoogerwerf et al. summarized that a regular whole-blood donation is associated with psychological and hormonal stress in a recent review [8]. One negative experience combined with whole blood donation leads to a significantly higher pre-donation blood pressure at the subsequent visit indicating an anticipatory stress reaction [9]. Psychological factors such as fear play a significant role in whether blood donation incidents occur. This often affects young first-time donors [10, 11].Vasovagal reactions are the most common adverse events during or after allogeneic blood donations. Minor symptomatic (presyncope) reactions result in up to 2.72% of donations [12], and major reactions with injury were reported in up to 0.09% of donations [13].

These adverse effects are stated to influence the retention rate of blood donors negatively [14, 15]. A decreased return rate by 34% of donors who have experienced a vasovagal reaction was reported by Newman and colleagues [16]. First-time donors with a negative experience have a lower return rate for a second blood donation and a higher risk for a vasovagal reaction until at least the third donation [17]. Previous studies have identified age, weight, body mass index, first-time donor, and predonation systolic and diastolic pressure as possible risk factors for vasovagal reactions [18–20].

Our study aimed to predict fainting in voluntary blood donors and to identify potential factors accounting for the occurrence of a vasovagal reaction during blood donation using modern machine learning algorithms. Other studies on machine learning focus on eligibility of donors [21] or use elaborate donor observation [22]. All vasovagal responses across the time course of donation were included in this study. Our focus was to establish a predictive model for categorizing the main influencing variables for this adverse reaction. In contrast to other studies, we included weather data from the place of blood donation in our analysis since it has been speculated for a long, that associated weather factors could essentially contribute to the rate of fainting [23–25]. The findings of our work should contribute to designing strategies to minimize these negative experiences and increase the retention rates for further donations.

## Materials and methods

Data from all whole blood and apheresis (thrombocytes, plasma, no erythrocyte apheresis) donors from January 2017 to December 2020 of the Red Cross blood donation service Linz were analyzed for the current study. Demographic characteristics (age, sex, blood type), donor-specific information (blood pressure, pulse, medical questionnaire), donation-specific data (donation procedure, donation site, association type), and weather data on the day of blood donation (temperature, humidity, dew point, barometric pressure, etc.) were used as features for seven different machine-learning algorithms (random forests (RFs), artificial neural networks (ANNs), X gradient boosting, adaptive boosting (XGBoost), k nearest neighbors (KNN), logistic regression (LOGREG), and support vector machines (SVM)). These data originated from standard procedures during blood donation; all records were fully anonymized. This retrospective study comprises only data of donors who met the acceptability criteria according to the Austrian Blood Donor Regulation [26] before being subjected to phlebotomy (Table 1) and if the donor or legal guardian(s) had given written informed consent for the provision of the data for research purposes (the study protocol was approved by the Ethics Commission of Upper Austria—Dec. Helsinki). In total 228 846 donations from 88 003 donors were used in this study. All donations were considered as individual blood donations, and no connection was drawn between multiple donations of one donor. Table 2 lists the characteristics of the blood donor population and their donation(s). 3090 (1.4%) of those donations were aborted due to fainting either before or after puncture and flagged by staff members according to a standardized procedure. The study includes only parameters that were known immediately before donation, therefore it does not consider the amount of blood already donated before fainting or predict the time of the donation when the vasovagal reaction will happen. There were 286 fainting cases that happened at the beginning of donation (< 100 mL), 1197 during the donation and 250 after the donation (> 400 mL).

**Table 1:** Acceptance criteria for blood donation at Red Cross Transfusion Service of Upper Austria

Criterion	
Age	≥ 18 years
Weight	≥ 50 kg
Pulse	50 ≥ pulse ≤ 100 bpm
Blood pressure (BP)	
Systolic	100 ≥ BP ≤ 180 mmHg
Diastolic	50 ≥ BP ≤ 100 mmHg
Temperature	
Male	≤ 37.5 °C
Female	≤ 38.0 °C
Haemoglobin	
Male	≥ 13.5 g/dL
Female	≥ 12.5 g/dL
Questionnaire medical history	No exclusion criterion according Austrian blood donor guidelines

**Table 2:** Donor and donation characteristics concerning fainting reactions

	All donations 228 846 (100%)	Donations without fainting 227,131 (99.25%)	Donations with fainting 1715 (0.75%)
Female	89,783 (39.2%)	88 876 (39.1%)	907 (52.9%)
Age (years)	40 ± 13	40 ± 13	26 ± 8
Height (cm)	174 ± 9	174 ± 9	172 ± 11
Weight (kg)	79 ± 16	79 ± 16	69 ± 16
BMI (kg/m^2^)	26 ± 4	26 ± 4	23 ± 3
Blood pressure (mmHg)	140/85	140/85	135/82
Body temperature (°C)	36.6 ± 0.4	36.6 ± 0.4	36.8 ± 0.4
Ambient temperature (°C)	12.6 ± 8.9	12.6 ± 8.9	12.4 ± 9.0
Dew point (°C)	6.4 ± 6.8	6.4 ± 6.8	6.4 ± 7.0
Humidity (%)	69 ± 17	69 ± 17	70 ± 17
Wind speed (km/h)	14 ± 9	14 ± 9	14 ± 9
Atmospheric pressure data (hPa)	1018 ± 8	1018 ± 8	1018 ± 9
Sunshine data (%)	48 ± 45	48 ± 45	48 ± 45
Blood group			
0	97 095 (42.4%)	96 368 (42.4%)	727 (42.5%)
A	93 878 (41.0%)	93 211 (41.0%)	667 (38.9%)
B	26 761 (11.7%)	26 540 (11.7%)	221 (12.9%)
AB	10 803 (4.7%)	10 710 (4.7%)	93 (5.4%)
No (valid) data	309 (0.1%)	302 (0.1%)	7 (0.4%)
Donation history			
First time	27 208 (11.9%)	26 129 (11.5%)	636 (37.1%)
Repeat	201 592 (88.1%)	200 956 (88.5%)	1079 (62.9)
No (valid) data donation	46 (0.0%)	46 (0.0%)	0 (0%)

### Statistics

For the prediction of fainting, we employed the model selection procedure for seven different, state-of-the-art machine learning methods: random forests [27], artificial neural networks, X gradient boosting, adaptive boosting, k nearest neighbors, logistic regression, and support vector machines (SVM). An implementation of a support vector machine that can handle unbalanced are potential support vector machines (PSVM) and was applied in this study [28].

Since our data set is heavily unbalanced we used “downsampling” for all applied algorithms. Downsampling utilizes the same amount of positive and negative cases for training. All donor data were obtained from electronic health records. The weather data were obtained from the Airport of Linz (LNZ) every day at noon.

The primary outcome of interest was the total number of blood donors who fainted during the donation procedure. This was registered by the attending nurse or the attending physician. After that, the data set underwent extensive data pre-processing and data cleaning. The data cleaning included the detection of typos and out-of-range values as well as the imputation of missing values:

All features with more than 25% of missing values were excluded, providing a save approach with little impact on model quality. The remaining missing values were imputed. We used the so-called “strawman imputation” and — in line with standard statistical and data reporting guidelines. Strawman imputation is defined as imputing using the median for missing values for continuous variables, and for missing categorical variables, the most frequently occurring non-missing value (ties are broken at random) [29].We also employed an advanced multi-imputation method, ‘missForest’, which is a machine learning-based data imputation algorithm that operates on the random forest algorithm. Both ways yielded equally good results hence we decided in favor of the more straightforward method. Censored numerical data were truncated (e.g., “ < 0.1” was replaced by 0.1). Categorical features with more than two values were one-hot encoded. Ordinal features were encoded as positive integers. Binary and numerical features were included as they were.

This resulted in a dataset with 92 variables and 228 846 blood donations from 88 003 donors for analysis.

We employed seven state-of-the-art machine learning methods: random forests, artificial neural networks, gradient boosting machines, adaptive boosting, k nearest neighbors, support vector machines, and logistic regression, using packages Amelia 1.7.6, Boruta 7.0.0, caret 6.0-86, readxl 1.3.1, ROCR 1.0-11, pROC 1.16.2, MLeval 0.3 and randomForest 4.6-14. A general overview of these methods is given in Saravanan et al. [30].

Data were split into training and test data sets. We applied a random search with 25 iterations for our hyperparameter selection (i. e., hyperparameter search) for all models except the ANNs where we used a grid search, both of which are provided by the “caret”-package for R (R 4.0.0, Vienna, Austria). For training models using each machine learning method, we used five-fold cross-validation on the training set. Finally, we used the test data set to assess each method’s generalization to previously unseen cases.

To evaluate the performance of our models, we used the following quality measures: positive and negative predictive value (PPV, and NPV, respectively), area under the receiver-operator characteristics curve (AUC), and the F1-Score. The PPV and NPV are the proportions of positive and negative results for given diagnostic tests that are true positive and true negative results, respectively. The AUC is the area under the receiver operating characteristic curve, a statistical parameter between 0.5 and 1.0, which describes the prediction quality of a model, with 0.5 being a random prediction and 1.0 a perfect prediction. The F1 score is the harmonic mean of precision and recall. We report these measures as our model was applied to a moderate to highly unbalanced setting where classification accuracy (ACC) would be of limited value.

Using the Boruta package of the R software package, we could determine the most essential features for predicting blood donation associated fainting using a random forest model. The algorithm uses a wrapper approach built around a random forest classifier. The algorithm is an extension of the idea introduced by Stoppiglia, Dreyfus, Dubois, and Oussar (2003) to determine relevance by comparing the relevance of the real features to that of the random probes [31, 32].

The code we used can be obtained from the authors upon request.

## Results

One thousand seven hundred fifteen fainting reactions (mild, moderate, and severe) were observed in 228 846 donations from 88,003 donors (prevalence rate of 0.75%) over the study period of 48 months. A detailed summary of all data used for analysis is given in Table 2. An overview of all donors who had fainting problems and did not have any issues is also listed in Table 2.

**Fig. 1:**
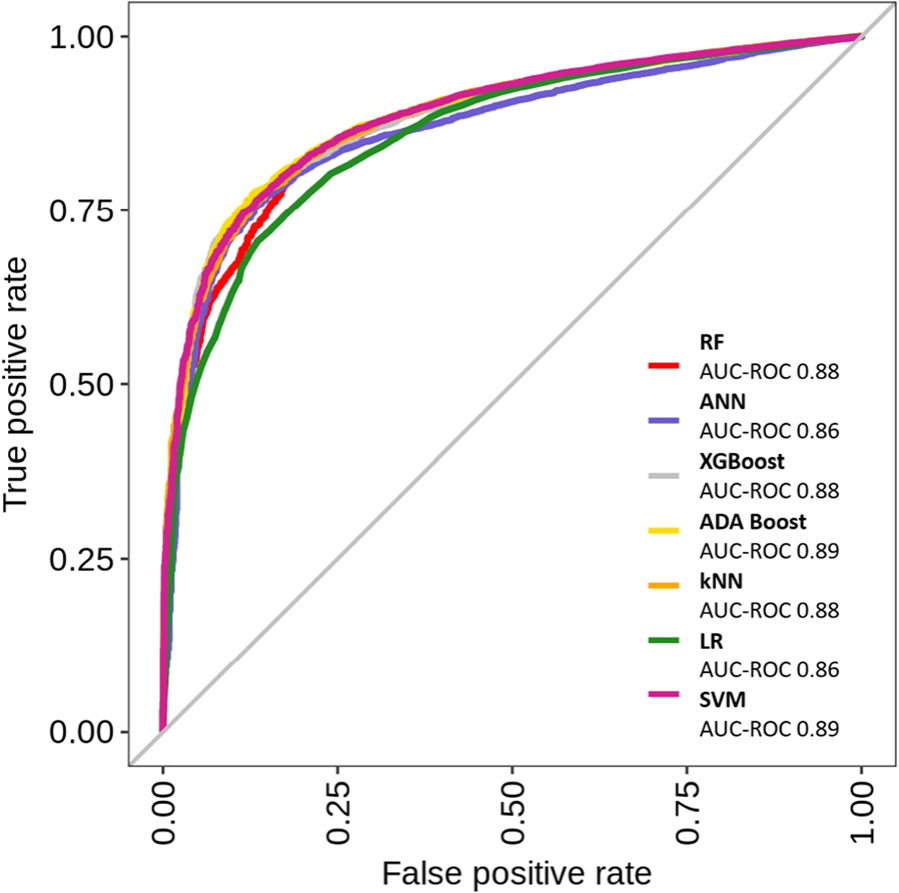
ROC curves of the seven machine learning models used. In the figure legend, the AUC of the ROC analysis is given for each of the models

All machine learning models yielded high AUCs in the ROC analysis, with values ranging from 0.86 (KNN) to 0.89 (XGB) (Fig. 1). Also, the F1-scores ranged from 0.855 (SVM) to 0.888 (RF) for all models investigated (Table 3), indicating a high capability of these models to predict fainting during blood donation despite the underlying problem being very asymmetric. The NPV was highest for the SVM with 0.999 and lowest for RF with 0.998. None of the models outperformed the other ones. Even the oldest model, the logistic regression, yielded comparable results.

**Table 3:** Model selection results for the seven machine learning methods

	PPV	NPV	AUC	F1-score
*All features*				
RF	***0.030***	0.998	0.88	**0.89**
ANN	0.028	0.998	0.86	0.86
XGB	0.026	0.998	0.88	0.87
ADA	0.028	0.998	***0.89***	0.87
LR	0.026	***0.999***	0.88	0.87
kNN	0.025	0.998	0.86	0.83
SVM	0.024	***0.999***	***0.89***	0.86

Bold italics indicate the best model for a given parameter

*PPV* positive predictive value, *NPV* negative predictive value, *AUC* area under the curve of the ROC analysis, *ACC* accuracy

The top-ranked features for prediction obtained with the Boruta package are the systolic and diastolic blood pressure, ambient temperature, relative humidity, the dew point, the atmospheric pressure, sunshine hours, wind-related data, gender, body weight, BMI, and height (Table 4). All other remaining features, especially questions from the questionnaire, are weighted considerably less.

**Table 4:** Feature importances obtained by the Boruta algorithm in arbitrary units

	Relative feature importance
Systolic blood pressure	41 (38–44)
Diastolic blood pressure	38 (30–45)
Ambient temperature	36 (34–39)
Relative humidity	33 (31–36)
Dew point	33 (30–35)
Atmospheric pressure	33 (31–35)
Percentage sunshine	28 (25–30)
Peek wind speed data	27 (25–30)
Peek wind direction data	25 (25–29)
Gender	23 (20–25)
Weight	23 (20–25)
BMI	23 (19–26)
Height	21 (20–22)
Wind direction data	12 (10–15)
Body temperature	10 (10–11)

Mean and 95% interval are given

## Discussion/conclusion

The main result of our study is that fainting reactions during blood donations can be predicted with similar good precision by seven mathematically different machine learning algorithms using the properties of the blood donor and local weather reports. Regardless of the algorithm used, the AUCs and F1 scores were close to 0.9, indicating the high potential of each of these algorithms for detecting donors at risk in our setting. However, it must be pointed out that although the negative predictive value of all of these models is relatively high, the positive predictive value is meager. For daily routine, this translates to a situation where a blood donor with a negative prediction can be reasonably sure, that no fainting will happen. In contrast, a person with a positive forecast will not necessarily faint during the donation procedure.

To our knowledge, this study is the first to predict fainting episodes using seven machine learning algorithms with a combination of donor-associated features and weather data. We obtained very high AUC- and F1 values for all models indicating the vast potential for using a variety of donor and weather data. This approach is readily applicable even for daily clinical practice since all necessary features for the prediction can be obtained in real-time from the questionnaire a donor has to fill in before blood donation and from a local weather station delivering the relevant weather data. However, those variables that are most capable of predicting the fainting are those, that are not as easy to obtain as from a questionnaire.

A correct and timely prediction of fainting episodes might enable one to improve the donation experience by making adequate preparations and monitoring patients at risks, such as pre-donation hydration or applied muscle tension [33].

The results of our machine learning prediction model showed that systolic and diastolic blood pressure are the two most essential features predicting fainting episodes. However, persons with highly elevated or shallow blood pressure were deferred from blood donation, so the data of these people were not included in our analysis. Hoogerwerf et al. published 2015 that those blood donors who experienced an adverse reaction during a whole blood donation had a significantly higher blood pressure before the donation process at the subsequent visit, indicating an anticipatory stress reaction in the following donation [9]. This finding that blood pressures are the most important predictors of donation complications somewhat contrasts with previous studies, where young age and undergoing first-time donation were the most essential predictors [34–36]. It is speculated that older donors are hemodynamically more stable [37], while younger people have the highest baroreflex sensitivity [38]. The stress before blood donation is much higher in first-time and young donors than experienced blood donors. From registration and eligibility assessment to phlebotomy, the whole procedure causes elevated psychological risk factors leading to vasovagal reactions due to an increase in pulse or arterial pressure. This leads to a vagal stimulation resulting in bradycardia and hypotension.

However, the seven most important features are all weather-associated parameters, indicating that ambient conditions might significantly predict donation-associated fainting. These parameters are also more important than height, weight, and gender, although these values also play a role in predicting fainting episodes. Blood donors' low weight and size correspond with a smaller blood volume and, therefore, higher fainting exposure. Weight as a risk factor of adverse vasovagal reactions was also found in previous studies [35, 36, 39–41]. Female sex was only a minor risk factor in our machine-learning algorithms that considered various confounding factors. This finding corresponded to Trouern-Trend et al. [36].

The influence of weather-associated features on fainting during blood donations has not been investigated thoroughly yet. It can be speculated that higher temperatures and specific constellations of barometric pressure and humidity might provoke fainting episodes in blood donors, but taking a look at our descriptive data (Table 2) does not yield too many insights. Although weather features are dominant in the feature importance analysis, one might recognize that no clear correlations can be seen in the descriptive statistics of these values. However, modern machine learning algorithms' strength is finding hidden correlations in a data set that cannot be recognized otherwise. Surprisingly the relative feature importance is very similar for all weather parameters, and associated weather features are an essential part of our final models.

We obtained very high AUC- and F1-values for all seven models indicating the vast potential of these algorithms for predicting fainting episodes in a clinical setting by some basic demographic parameters, the questionnaire every blood donor had to fill in before the donation procedure, and the weather data. Since these data are available for each blood donation at our institution, we believe that integration of this approach is feasible in our setting and should be easily achievable for other locations. The correct prediction of fainting episodes might improve the donation experience by preparing and monitoring patients at risk.

Due to our problem’s asymmetrical nature, we could only obtain a relatively low positive predictive value using the features we got. Furthermore, our positive results for the negative predictive value have to be seen in the light of the asymmetry of the underlying problem. A trivial classifier (“no fainting expected”) might result in a negative predictive value in our example of 0.9925, which can be calculated as the ratio of non-fainting patients over all patients. In other words, between 7 and 8 donors will be misclassified as not fainting. Our best negative predictive value obtained by LR and an SVM was 0.999, indicating misclassification in only 1 per 1000 donors. Although this sevenfold improvement in identifying non-fainting blood donors is impressive from a mathematical point of view, the clinical significance has to be assessed by potential users.

One limitation of our study was the lack of discrimination between mild and moderate fainting episodes. This deficit was attributed to the fact that we only differentiate routinely between severe and other adverse vasovagal reactions during blood donation. Mild reactions might less influence blood donors, whereas more severe reactions might endanger donors. However, since severe fainting episodes are relatively rare, we believe the correct prediction of these episodes is challenging.

In summary, prediction models with machine-learning algorithms can be helpful in reducing negative experiences during blood donation and contribute to improving donor safety. Using modern machine learning algorithms, it is possible to identify blood donors that will have no vasovagal reaction through the donation procedures if donors’ properties and weather data are used. The clinical applicability of this approach is high, but the positive net effect of such screening should be investigated in a prospective clinical study.

## Data Availability

The data that support the findings of this study are available from the Red Cross Upper Austria, but restrictions apply to the availability of these data, which were used under license for the current research, and so are not publicly available. However, data are available from the corresponding author upon reasonable request and with permission of the Red Cross Upper Austria.
